# Determinants of growth monitoring and promotion service utilization among children 0–23 months of age in northern Ethiopia: unmatched case-control study

**DOI:** 10.1186/s40795-021-00470-y

**Published:** 2021-11-08

**Authors:** Samuel Dagne, Jemal Aliyu, Yonatan Menber, Yosef Wassihun, Pammla Petrucka, Netsanet Fentahun

**Affiliations:** 1 Department of Nutrition and Dietetics, School of Public Health, College of Medicine and Health Sciences, Bahir dar University, Bahir dar, Ethiopia; 2 Department of Health Promotion and Behavioral Sciences, School of Public Health, College of Medicine and Health Sciences, Bahir dar University, Bahir dar, Ethiopia; 3 University of Saskatchewan, Saskatoon, SK Canada

**Keywords:** Growth monitoring and promotion, Under two years’ children, Legambo District, Northern Ethiopia

## Abstract

**Background:**

One of the strategies to promote child health and reduce child mortality is growth monitoring and promotion services. But, there is limited information on determinants of Growth Monitoring and Promotion service utilization.

**Objective:**

To identify determinants of growth monitoring and promotion (GMP) service utilization among children 0–23 months of age in Legambo district, South Wollo zone, Northern Ethiopia, 2020.

**Methods:**

Community based un-matched case-control study was conducted on 363 (91 cases and 272 controls) study participants from March 15 to April 15, 2020. A multi-stage sampling technique was employed to select the study participants. Bivariable and multivariable logistic regressions were performed and an adjusted odds ratio with 95% confidence intervals was estimated to identify determinants of GMP service utilization.

**Results:**

A total of 358 mothers (89 cases and 269 controls) with 98.6% response rate were included in the study. The mean (±SD) age of child was 11.66(±6.29) months among controls and 15.02 (±6.06) months among cases. Good maternal knowledge (AOR) = 2.42; 95% CI: 1.23, 4.75), favorable attitude (AOR = 2.45; 95% CI; 1.20, 4.98), counseling on GMP (AOR = 2.34; 95% CI; 1.19, 4.56), attending ante natal care services (AOR = 2.46; 95% CI: 1.18, 5.16), index child age 12–17 months (AOR = 3.45; 95% CI: 1.26, 9.41) and 18–23 months (AOR = 4.38; 95% CI: 1.53, 12.49), and short distance to health facilities (AOR = 4.53; 95% CI; 1.99, 10.28) were determinants of GMP service utilization.

**Conclusion:**

Index child age, good knowledge, favorable attitude, attending antenatal care services, receiving nutritional counseling, and a short distance to health facility were determinants of GMP service utilization. Nutritional interventions should emphasize nutritional counseling and accessibility of growth monitoring and promotion services.

**Supplementary Information:**

The online version contains supplementary material available at 10.1186/s40795-021-00470-y.

## Introduction

Child malnutrition is one of the world’s serious public health problems. Around 45% of deaths among children under 5 years of age are linked to undernutrition [1]. Worldwide, 151 million under-5 years of age children were stunted, 51 million were wasted, and 52 million were overweight [2]. Child malnutrition is also still a high public health problem in Ethiopia. According to the 2019 Ethiopian mini Demographic and Health Survey (EDHS) report, the prevalence of stunting was 37%, underweight 21%, and wasting 7% [3]. Similarly, the prevalence of stunting in the Amhara region was 41% which was the third-highest from the regions of the country [3].

Poor child growth and development in the early stages of life can increase the risk of infections, morbidity, and mortality together with decreased mental, cognitive and economic development [4–6]. The promotion of child growth and development is one of the health priorities to control child mortality and poverty reduction [7]. Even though Growth Monitoring and Promotion (GMP) is one of the prerequisites for good child health, several studies showed that there is a big difference between the purpose and the practice of GMP [8, 9]. The high prevalence of malnutrition in many low and middle-income countries including Ethiopia supports this fact [3, 10, 11]. Participation in GMP remains relatively low in Ethiopia. Research findings from Southern Ethiopia showed that the coverage of GMP service utilization was16.9% in the Mareka district [12], and 11% in Butajira [13].

Previous studies reported that socio-demographic, maternal, and health professional-related factors influence GMP service utilization [12–18].

The National Nutritional Program (NNP) of Ethiopia considers GMP as one of the strategies for improving the nutritional status of the children and has been implementing at the community level through health extension programs. GMP is a prevention activity comprised of GM linked with a promotion that increases awareness about child growth; improves caring practices; increases demand other services, as needed; and serves as the core activity in an integrated child health and nutrition program [9]. Promoting and improving child health during the window of opportunity period starting from conception to a child’s second birthday, is crucial for survival [19].

Even though the Ethiopian government has been implementing GMP services at a community level, the available data indicated that malnutrition is still high and participation in growth monitoring remains relatively low in the country. There is limited evidence on determinants of Growth Monitoring and Promotion service utilization in Ethiopia. This study aimed to identify determinants of GMP utilization among children less than 2 years in Legambo District, South Wollo zone, Northern Ethiopia. Therefore, identifying the determinants of low coverage of GMP service utilization helps nutrition program implementers to design evidence-based GMP interventions.

## Methods and materials

### Study area and period

The study was conducted from March 15 to April 15, 2020, in Legambo District, South Wollo zone, Northern Ethiopia. The district is found in the South Wollo zone Amhara region. it is situated on the beautiful highlands of south Wollo at an altitude of about 3000 m above sea level and is located 100 km to Dessie (the capital city of South Wollo zone), 430 km from Bahir Dar (the Capital city of the Amhara region) and 501 km far from Addis Ababa (the Capital city of Ethiopia). The district has 33 health Posts, 9 health centers, 1 hospital, 78 Health extension workers. The total population of the district was 281,974 with 147,160 males and 134,748 females while the total number of children with the age of o month to 23 months was 10,172 in the year 2017 which was projected from the Woreda Administration office [20].

### Study design

A community-based unmatched case-control study design was employed.

### Eligibility criteria

All mother-child pairs with 0–23 months residing in the Legambo district during the study period were included in the study. Whereas, children with the age of 0 to 23 months suffering from chronic illnesses and those on treatment such as TB, HIV were excluded from the study.

### Sample size determination

The sample size was calculated using Epi Info version 7.2.1.1 by considering the following assumptions: proportion of reach wealth status who utilized GMP service was 19.5% among controls and 5% among cases from the study conducted in Southern Ethiopia, 5% type I error, 80% power, 1:3 cases to controls ratio, design effect of 1.5 and 10% non-response **[**12]**.** The final sample size was 363(91 cases and 272 controls).

### Sampling procedures

A multi-stage sampling technique was used to select the study participants. Out of 34 kebeles in the district, 7 kebeles were selected using the lottery method. The list of mother-child pairs aged 0–23 months and their house numbers from each kebele were obtained from the health extension workers. House to house censuses was made to identify cases and controls and children aged 0–23 months were identified and registered sequentially and had got identification number as case and control. And then, the total sample size was allocated proportionally to each kebele. Finally, both cases and controls were selected by a simple random sampling technique.

### Operational definitions

**Case:** Participation of a child for GMP services at least once for 0 months, at least two times for 1–3 months, at least five times for 4–11 months, and at least four times per year for 12–23 months.

**Control**: a child who had not participated in GMP services at least once for 0 months, at least two times for 1–3 months, at least five times for 4–11 months, and at least four times per year for 12–23 months.

**Good knowledge:** is defined as scored above 7 from the total ten knowledge questions [13].

**Poor knowledge:** is defined as scored below 7 were considered as having poor knowledge [13].

**Unfavorable attitude:** is defined as a score of < 75%.

**A favorable attitude:** is defined as a score of ≥75% [13].

### Data collection tools and procedures

The data were collected using an interviewer-administered structured questionnaire. The questionnaire includes socio-demographic, economic, health care, behavioral factors, and maternal/caregiver’s related characteristics and adapted from previous studies [12, 13, 16, 21, 22] and collected by well trained and experienced two clinical nurses and three diploma midwives and three health officer supervisors.

ANC visit was assessed based on the minimum recommended visits (yes; for having four or more visits and no; for less than four visits). And, PNC was also assessed based on the minimum recommended visits (yes; for having at least one visit in the post-partum period and no; for not visits at all). The vaccination status of children was checked by observing the immunization card and if not available mothers /caregivers/ were asked to recall it. BCG vaccination was checked by observing a scar on right (also left) arm. The wealth index of households was determined using the Principal Component Analysis (PCA) by considering latrine, water source, household assets, livestock, and agricultural land adopted from EDHS 2016 [10]. The responses of all variables were classified into two scores. The highest score was coded as 1 and the lower score was given code 0. Assumptions of PCA were checked to carry out the wealth index score. In PCA to determine the number of components that would retain, eigenvalue-one criterion was used and those variables having a commonality value of greater than 0.5 were used to produce factor scores. Lastly, the score for each household on the first principal component was retained to create the wealth score. Finally, tertials of the wealth score were created to categorize households as poor, medium, and rich.

Distance to health facility determined by the distance (time taken to reach the health facility from mothers’ home to the nearest health facility). Distance to health facility was classified as less than 1 h and more than 1 h to reach the nearest health facility [23]. Knowledge of mothers towards GMP service utilization was assessed using ten knowledge questions. Each questions has two response (yes = 1 or 0 = no). The total score ranges from 0 to 10. A score above 7 was categorized as good knowledge and below 7 was categorized as poor knowledge [13]. The attitude of the mother to GMP service was assessed by 12 attitude questions using Likert scale measures (1 = strongly disagree, 2 = disagree, 3 = neutral, 4 = agree and 5 = strongly agree). The total score ranges from 12 to 60. A score of ≥75% was categorized as favorable attitude and a score of < 75% was categorized as unfavorable attitude [13].

### Data quality assurance

The questionnaire was translated to the Amharic language and translated back to English to ensure consistency. The questionnaire was pre-tested in 5% of the sampled population in non-selected kebeles before the actual data collection. Data collectors and supervisors were trained for 2 days. Test-retest reliability of the research instrument was established during pretesting. Test re-test reliability was established by examining the consistency of pre-test responses. On spot-checking and corrections were made for incomplete questionnaires by the supervisor. The overall data collection process was controlled by the principal investigator.

### Data processing and analysis

The data were coded and entered and into Epi info version 7 and exported to SPSS version 23 for analysis. Descriptive statistics were computed and presented using tables, figures, and charts. Model goodness of fitness was assessed by using Hosmer and Lemeshow test. Multi-colinearity between independent variables was checked. Bi-variable logistic regression was executed and variables with *p* < 0.25 were fitted to the final multivariable logistic regression to adjust for potential confounders. In the final model, variables with a *P*-value < 0.05 and AOR of 95% CI were considered to declare the statistical significance and the strength of association.

## Results

### Socio-economic characteristics of study participants

A total of 358 mothers (89 cases and 269 controls) with a 98.6% response rate were included in the study. The mean age of mothers was 27.28 with an SD of ±5.074. The average family size was 4.4 with an SD of ±1.50. About 55(61.8%) and 150(55.8%) of respondents were farmers among case and controls, respectively (Table 1).

**Table 1:** Socio-economic characteristics of mothers/caregivers in Legambo district, South Wollo zone, Northern Ethiopia, 2020

Variable	Category	Case: ***n*** = 89 (%)	Control: ***n*** = 269 (%)
**Maternal age**	15–30	58 (65.2)	180 (66.9)
	30–49	31 (34.8)	89 (33.1)
**Marital status**	Married	89 (100)	263 (97.8)
	Unmarried^a^	0	6 (2.2)
**Maternal education**	Cannot read and write	26 (29.2)	132 (49.1)
	Can read and write	2 (2.2)	5 (1.9)
	Primary school	31 (34.8)	94 (34.9)
	Secondary and above	30 (33.7)	38 (14.1)
**Husband education**	Cannot read and write	25 (28.1)	133 (49.4)
	Can read and write	5 (5.6)	22 (8.2)
	Primary	26 (29.2)	75 (27.9)
	Secondary and above	33 (37.1)	39 (14.5)
**Occupation of mother**	Housewife	22 (24.7)	99 (36.8)
	Farmer	45 (50.6)	150 (55.8)
	Merchant	11 (12.4)	12 (4.5)
	Employed	11 (12.3)	8 (3.0)
**Husband occupation**	Farmer	55 (61.8)	216 (80.3)
	Merchant	8 (9.0)	28 (10.4)
	Employed	26 (29.2)	17 (6.3)
	Others*	0	8 (3.0)
**Wealth index**	High	37 (41.6)	84 (31.2)
	Medium	27 (30.3)	95 (35.3)
	Low	25 (28.1)	90 (33.5)
**Family size**	< 4	60 (67.4)	162 (60.2)
	≥4	29 (32.6)	107 (39.8)
**Birth order**	1	37 (41.6)	94 (34.9)
	≥2	52 (58.4)	175 (65.1)

^*^Daily laborer and students

^a^widowed and divorced

### Growth monitoring and promotion service and child age

**Fig. 1:**
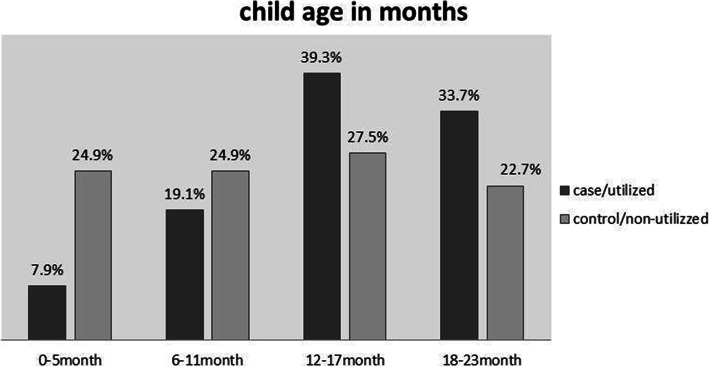
Growth Monitoring and Promotion service and child age in Legambo district, South Wollo zone, Northern Ethiopia, 2020

About 35 (39.3%) and 74 (27.5%) of children in the age group of 12–17 months age utilized GMP services in both cases and controls, respectively. Only 7(7.9%) of children within the age group of 0–5 months of age utilize GMP service among cases (Fig. 1).

### Maternal knowledge and attitude to GMP service

The median knowledge and attitude scores among cases were 10 (IQR = 10) and 51(IQR = 25), respectively. The median knowledge and attitude scores among controls were 14(IQR = 10) and 44 (IQR = 29), respectively. Around 72(80.9%) of cases and 133(49.4%) of the controls group had good knowledge. Regarding the attitude; (83.1% of cases and 48% of controls) had a favorable attitude. About 16 (18%) of cases and 32% of controls don’t know when the growth monitoring service is started (Table 2).

**Table 2:** knowledge and attitude of mothers/caregivers towards GMP service utilization in Legambo district, South Wollo zone, Northern Ethiopia, 2020

Variable		Case: ***n*** = 89 (%)	Control: ***n*** = 269 (%)
Heard about GMP	Yes	87 (97.8)	194 (72.1)
	No	2 (2.1)	75 (20.9)
Know the age groups for GMP	Yes	77 (86.5)	193 (75.4)
	No	12 (3.4)	76 (21.2)
Know the starting time for GMP	Yes	73 (82)	183 (68)
	No	16 (18)	86 (32)
Know interval between GMP	Yes	82 (92.1)	175 (65.1)
	No	7 (7.9)	94 (34.9)
Know who to perform GMP	Yes	84 (94.4)	182 (67.7)
	No	5 (5.6)	87 (32.3)
Know place of GMP services	Yes	85 (95.5)	191 (71)
	No	4 (4.5)	78 (29)
GMP service has a benefit for the child	Yes	88 (98.9)	222 (82.5)
	No	1 (1.1)	47 (17.5)
Know when the growth chart is flattening	Yes	70 (78.7)	135 (50.2)
	No	19 (21.3)	134 (49.8)
Know when the growth chart is rising	Yes	70 (78.7)	134 (49.8)
	No	19 (21.3)	135 (50.2)
Know when the growth chart is falling	Yes	70 (78.7)	133 (49.4)
	No	19 (21.3)	136 (50.6)
Knowledge	Good	72 (80.9)	133 (49.4)
	Poor	17 (19.1)	136 (30.6)
Attitude	Favorable	74 (83.1)	129 (48)
	Unfavorable	15 (16.9)	140 (52)

### Health care service-related characteristics

About 74(83.1%) mothers in the cases group (utilized) and 132(49.1%) mothers/caregivers in the controls group (Non- utilized) had 4 and above antenatal care (ANC) visits. The majority of mothers/caregivers in both cases group (67.4%) and controls group (71%) gave birth (for this baby) in health institutions. Only 70(78.7%) mothers in case groups and 123(45.7%) mothers in the control group were counseled on GMP (Table 3).

**Table 3:** Health care service utilization-related characteristics of mothers/caregivers in Legambo district, South Wollo zone, Northern Ethiopia, 2020

Variable	Categories	Case: ***n*** = 89 (%)	Control: ***n*** = 267 (%)
Place of delivery	Home	29 (32.6)	78 (29)
	Health institution	60 (67.4)	191 (71)
Immunization	Fully immunized	86 (96.6)	262 (97.4)
	Not immunized	3 (3.4)	7 (2.6)
ANC utilization	Yes	74 (83.1)	132 (49.1)
	No	15 (16.9)	137 (50.9)
PNC utilization	Yes	68 (76.4)	117 (43.5)
	No	21 (23.6)	152 (56.5)
Counseling on GMP	Yes	70 (78.7)	123 (45.7)
	No	19 (21.3)	146 (54.3)
Service quality	Good	51 (57.3)	127 (47.2)
	Poor	38 (42.7)	142 (52.8)
Distance to health facility	< 1 h	79 (88.8)	154 (57.2)
	≥1 h	10 (11.2)	115 (42.8)
Mode of delivery	SVD	85 (95.5%)	257 (95.5%)
	Instrumental	0	5 (2.6)
	C/section	4 (4.5)	7 (4.5)
Counseled on nutrition	Yes	88 (98.9)	32 (11.9)
	No	1 (1.1)	237 (88.1)
Vitamin A supplementation	Yes	86 (96.6)	45 (16.7)
	No	3 (3.4)	224 (83.3)

### Determinants of GMP service utilization

In the Bivariable regression analysis, child age, paternal education, maternal education, antenatal care utilization, counseling on GMP service utilization, postnatal care utilization, knowledge, attitude, service quality, access to media, and distance to health facility were associated with the GMP at *p*-value < 0.25. Finally, variables with a p-value < 0.25 were transferred to multivariable logistic regression and child age, knowledge, attitude, counseling on GMP, ANC service utilization and distance to health facility were independent predictors of GMP service utilization at *p*-value< 0.05. Mothers/caregivers who had good knowledge were 2.4 times more likely to utilize GMP service compared to mothers/caregivers who had poor Knowledge (AOR) = 2.42; 95% CI: 1.23, 4.75). Mothers/caregivers who had received counseling on GMP service were 2.3 times more likely to utilize GMP service compared to mothers/caregivers who had not received counseling (AOR = 2.34, 95% CI: 1.19, 4.56). Mothers/caregivers who reached nearby the health facility less than one hour were 4.5 times more likely to utilize GMP service as compared to mothers/caregivers who reached greater than one hour (AOR = 4.53; 95% CI: 1.99, 10.28).

Mothers/caregivers who had utilized ANC services were 2.46 times more likely to utilize GMP services as compared to mothers/caregivers who had not utilized ANC services (AOR = 2.46; 95% CI: 1.18, 5.16). Mothers/caregivers/ who had favorable attitudes were 2.45 times more likely to utilize GMP services as compared to unfavorable attitudes (AOR = 2.45; 95% CI: 1.20, 4.98). Mothers who have children age group between 12 and 17 months were 3 times and 18-23 months age groups were 4 times more likely to utilize GMP services as compared to who have Children with 0–5 age groups (AOR = 3.45; 95% CI: 1.26, 9.41) and (AOR = 4.38; 95% CI: 1.53, 12.49), respectively (Table 4).

**Table 4:** Predictors of GMP services utilization among children 0–23 months of age in Legambo district, South Wollo zone, Northern Ethiopia, 2020

N	Categories	Cases	Controls	95% confidence interval	
				COR	AOR
**Maternal education**	Illiterate	26 (29.2%)	132 (49.1%)	1	1
	Read and write	2 (2.2%)	5 (1.9%)	2.03 (0.37–11.04)	1.32 (0.19–9.17)
	Primary	31 (34.8%)	94 (34.9%)	1.67 (0.93–3.00)	1.29 (0.52–3.22)
	Secondary and above	30 (33.7%)	38 (14.1%)	4.01 (2.12–7.58)	1.96 (0.68–5.69)
**Partner education**	Illiterate	25 (28.1%)	133 (49.4%)	1	1
	Read and write	5 (5.6%)	22 (8.2%)	1.21 (0.42–3.49)	0.89 (0.23–3.50)
	Primary	26 (29.2%)	75 (27.9%)	1.84 (0.99–3.42)	1.25 (0.49–3.19)
	Secondary and above	33 (37.1%)	39 (14.5%)	4.5 (2.39–8.46)	1.87 (0.68–5.13)
**Child age in months**	0–5	7 (7.9%)	67 (24.9%)	1	1
	6–11	17 (19.1%)	67 (24.9%)	2.43 (0.95–6.24)	2.67-(0.91–7.85)
	12–17	35 (39.3%)	74 (27.5%)	4.53 (1.89–10.87)^*^	3.45 (1.26–9.41)^*^
	18–23	30 (33.7%)	61 (22.7%)	4.70 (1.93–11.49)^**^	4.38 (1.53–12.49)^**^
**Maternal knowledge**	Good	72 (80.9%)	133 (49.4%)	4.33 (2.42–7.73)^*^	2.42 (1.23–4.75)^*^
	Poor	17 (19.1%)	136 (30.6%)	1	1
**Maternal attitude**	Favorable	74 (83.1%)	129 (48.0%)	5.35 (2.92–9.79)^*^	2.45 (1.20–4.98)^*^
	Unfavorable	15 (16.9%)	140 (52.0%)	1	1
**Counseling on GMP**	Yes	70 (78.7%)	123 (45.7%)	4.37 (2.49–7.66)^*^	2.34 (1.19–4.56)^*^
	No	19 (21.3%)	146 (54.3%)	1	1
**Access to media**	Yes	52 (58.4%)	114 (42.4%)	1.91 (1.18–3.11)^*^	0.92 (0.48–1.77)
	No	37 (41.6)	155 (57.6%)		
**Distance to health facility**	< 1 h	79 (88.8%)	154 (57.2%)	5.89 (2.93–11.89)^**^	4.53 (1.99–10.28)^**^
	> 1 h	10 (11.2%)	115 (42.8%)	1	1
**ANC service utilization**	Yes	74 (83.1%)	132 (49.1%)	5.12 (2.79–9.37)^*^	2.46 (1.18–5.16)^*^
	No	15 (16.9%)	137 (50.9%)	1	1
**PNC service utilization**	Yes	68 (76.4%)	117 (43.5%)	4.20 (2.44–7.26)^*^	1.89 (0.95–3.62)
	No	21 (23.6%)	152 (56.5%)	1	1

^*^P-value < 0.05; ^**^ *P*-value < 0.001, *COR* Crude odds ratio, *AOR* Adjusted odds ratio

## Discussions

This study aimed to identify determinants of Growth monitoring and promotion service utilization using an unmatched case-control study among less than two years children and the study will generate information for the Ministry of Health and other organizations working in the child survival programs to design interventions to improve the activities of GMP.

The study pointed out that determinants of growth monitoring and promotion service utilization were index child age, maternal knowledge, maternal Attitude, utilization of ANC services, getting counseling about GMP, and distance to reach the nearest health facility.

Mother who had adequate knowledge of growth monitoring was more likely utilizes GMP than mother who had inadequate knowledge. A similar finding was reported from the study done in Areka town, Butajira, Kenya, and Ghana [13, 15, 21, 22]. This can be explained by the mother with adequate knowledge may able to understand the information displayed on the growth chart and that motivates to utilize GMP session.

In this study, a child in the age group of 6–11 months and 12–23 months were found that more likely to utilize GMP services as compared to infants in the age group of 0–5 months. This finding is similar to a study done in Southern Ethiopia Mareka district [12] and Butajira [21]. This could be explained in Ethiopian culture mothers perceived that taking their child to the GMP session will expose their child to the “evil eye”. Exposing children in front of people until they started walking is not accepted by the mothers [24]. These attitudes cause some mothers to be reluctant to attend the GMP session. Therefore, the GMP uptake may be improved in the first year of life considering the local cultural beliefs [25].

This study showed that mothers/caregivers who had favorable attitudes were more likely to utilize than those who had unfavorable attitudes. This finding was supported by a study on Areka town, southern Ethiopia [13]. The reason for this might be a good attitude of mother’s leads to happy to bring their child to the GM visits and this helps to utilize GMP session and for unfavorable attitude, one qualitative study conducted Loko Abaya District, Southern Ethiopia [24] showed that Mothers mentioned that they understood GMP as being used only for unhealthy (especially wasted) children. If their children are healthy and well-fed, they did not want to attend the GMP program.

According to this study mothers who utilized ANC services were more utilize GMP services than those who had not ANC service utilization. The possible justification for this may be during Antenatal care nutritional counseling is given and most of the mothers understand the service that was got from the health institution and after delivery, the mothers will be happy to attend the GMP session. This result is different from a study done in the Mareka district [12] showed that there is no significant association between the utilization of ANC services and GMP service utilization. This difference is may be due to study design; the previous study was used cross-sectional while this study was used case-control study design and time difference, at this time the coverage of ANC is increased.

This study identified that mothers/caregivers who received counseling about growth monitoring and promotion were more likely to utilize GMP services than those who did not receive counseling. This study is in line with a study in Kenya [15] which showed mothers/caregivers who received nutrition advice alongside GM services were more likely to participate in continued. The reason for this may be counseling has a greater impact on motivating mothers to attend GMP sessions.

In this study, mothers who traveled less than an hour to get to the nearest health facility from their home were more likely to utilize GMP services for those who travel more than one hour to get to the nearest health facility. This finding is similar to study in Southern Ethiopian, Mareka district [12] and also supported by the study in Kenya [15] showed that distance from respondent’s home to the facility 5 km; return journey were significantly associated with continued GM and similarly in Ghana [22] stated that distance between caregivers home and the child welfare clinic is a determining factor in child welfare clinic (CWC) attendance. The possible justification for this may be due to long distances to the health facilities may be a hindrance to the mothers to continue with growth monitoring especially if the children are looking well because of the competing roles. Socio-economic variables included in this study (marital status, occupation of mother, family size of the household, and wealth index) were not significantly associated with GMP service utilization. This might be due to the similar nature or living standard of mother was included in the study. Majority of mothers were farmers, married and lived in similar setting. As a limitation, there might be a recall bias while assessing the growth chart knowledge of the mothers, utilization of ANC service, and PNC services.

In this study, both maternal and partner education at the secondary level and above are significant in the unadjusted model. Despite the lack of statistical significance at the adjusted model, the maternal and paternal literacy /education seemed to show a trend in uptake of services with higher levels of education.

## Conclusion

Child age, good knowledge towards growth monitoring, favorable attitude towards growth monitoring and promotion, utilization of ANC services, counseling towards growth monitoring and promotion, and time to reach the nearest health facility within one hour were determinants of GMP service utilization. Nutritional interventions should emphasize nutritional counseling, utilization of ANC service, and accessibility of growth monitoring and promotion service to improve growth monitoring and promotion service utilization and reduce malnutrition in Ethiopia. It is also recommended that GMP services should be culturally sensitive.

## Supplementary Information

**Additional file 1.** Questionnaire_ English version.

## Data Availability

The datasets generated and/or analysed during the current study are not publicly available due to limitations of ethical approval involving the patient data and anonymity but are available from the corresponding author on reasonable request.

## Abbreviations

ANC: Ante Natal Care
AOR: Adjusted Odd Ratio
CSA: Central Statistics Agency
CI: Confidence Interval
DSS: Demographic Surveillance System
EDHS: Ethiopian Demographic Health Survey
GM: Growth Monitoring
GMP: Growth Monitoring and Promotion
PNC: Post Natal Care
SD: Standard Deviation
