# Supplementary material for: Determinants of growth monitoring and promotion service utilization among children 0–23 months of age in northern Ethiopia: unmatched case-control study
Source: BMC Nutr. 2021 Nov 8;7:67. doi: 10.1186/s40795-021-00470-y (PMC8573945; doi:10.1186/s40795-021-00470-y)
Supplement: Supplementary file 1 — Additional file 1. Questionnaire_ English version. [file 40795_2021_470_MOESM1_ESM.docx]

**Informed consent**

The above information regarding my participation in the study is clear to me. I have been given a chance to ask questions and my questions have been answered to my satisfaction. My participation in this study is entirely voluntary. I understand that my records will be kept private and that I can leave the study at any time. I understand that I will still get the same medical care whether I decide to leave the study or not and my decision will not change the care I will receive from medical centers.

Respondent agree to participate? YES NO

1. If yes, continue the interview
2. If no, skip to the next participant by writing reasons for her refusal.

______________________________________________________________________________ Informed consent Certified by

Relation of the respondent to the child________________________________________

Respondent’s signature/thumb print____________________ Date _______________________ Interviewer: Name _________________ Signature_____________

Questionnaire ID number ________________________

Date of interview ____________ Time started __________ Time completed__________

Checked by: Supervisor: Name ____________________________ Signature_____________

**Questionnaire (English version)**

Survey questionnaire to identify determinants of growth monitoring and promotion service utilization.

Questionnaire No__________ Kebele name _______House number__________

Growth Monitoring and Promotion service given based on the Guideline

1. Yes

2. No

| Part 1-child bio data | | | | | | | | | | | | | | |
| --- | --- | --- | --- | --- | --- | --- | --- | --- | --- | --- | --- | --- | --- | --- |
| S.no | Questions | | | Response | | | | | | | | Skip | | |
| 101 | Sex of the child | | | 1. Male  2. female | | | | | | | |  | | |
| 102 | Date of birth of the child | | | __________________ | | | | | | | |  | | |
| 103 | Child age in month | | | __________________ | | | | | | | |  | | |
| 104 | Place of delivery | | | 1.home  2.health center  3.Hospital  4.other | | | | | | | |  | | |
| 105 | Mode of delivery | | | 1.Viginal delivery  2.Cesarean section  3.Instrumental delivery  4.other | | | | | | | |  | | |
| 106 | How many times had the child received the basic immunization? (NB-see at the immunization card if available if not ask the mother recall) | | | __________________ | | | | | | | |  | | |
| 107 | Is GMP is given for the child | | | 1.yes  2.no | | | | | | | |  | | |
| 108 | If yes how many times | | |  | | | | | | | |  | | |
| Part 2-Socio demographic and economy factors | | | | | | | | | | | | | | |
| S.no | | Questions | | | Response | | | | | | | | Skip | |
| 201 | | Age of the mother in completed year? | | | __________________ | | | | | | | |  | |
| 202 | | What is your religion? | | | 1. Orthodox  2. Muslim  3. Catholic  4. Protestant  5.Others(specify)__________________ | | | | | | | |  | |
| 203 | | What is your ethnicity? | | | 1. Amhara  2. Oromo  3. Tigre  4. Guragie  5.Others( specify)_________________ | | | | | | | |  | |
| 204 | | Level of education | | | 1. cannot read and write  2. can read and write  3. Primary  4. Secondary  5.college and above  6.Other(specify)_______________________ | | | | | | | |  | |
| 205 | | What is your occupational status?  NB. more than one answer is possible | | | 1. House wife  2. Farmer  3. Merchant  4. Government employee  5. Private employee  6.Student 7.Others(specify)_____________________ | | | | | | | |  | |
| 206 | | What is your current marital status? | | | 1.Married  2.single  2. Divorced  3. Widowed | | | | | | | |  | |
| 207 | | What is the occupation of your husband? | | | 1. Farmer  2. Merchant  3. Government employee  4. Private employee  5. Daily laborer  6. Student  7. Others( specify)__ | | | | | | | |  | |
| 208 | | What is the educational status of your husband? | | | 1. cannot read and write  2. can read and write  3. Primary  4. Secondary  5.college and above  6. other(specify)_____________________ | | | | | | | |  | |
| 209 | | household family size | | |  | | | | | | | |  | |
| 210 | | Birth order | | |  | | | | | | | |  | |
| 211 | | household average monthly income | | | ______________birr | | | | | | | |  | |
| 212 | | Source of water supply | | | 1. Piped water  2. Protected spring  3. other (specify ________________ | | | | | | | |  | |
| 213 | | How long does it take to go there, get water and comeback? | | | _________________hour | | | | | | | |  | |
| 214 | | Do you have toilet your own toilet? | | | 1.yes  2.no | | | | | | | |  | |
| 215 | | Source of energy to cook | | | 1. Electric  2. Kerosene and wood  3. other (specify ______________ | | | | | | | |  | |
| 216 | | Main material of the roof? | | | 1. Grass and wood  2. Corrugated iron/metal  3. Other(specify_______________________ | | | | | | | |  | |
| 217 | | Main material of the floor? | | | 1. Sand  2.Cement and marble  3 Other (specify________________________ | | | | | | | |  | |
| 218 | | Main material of the walls? | | | 1. Wood with mud  2.Cement with stone  3.Other(specify________________________ | | | | | | | |  | |
| 219 | | Which one of the following found in your house?  Electricity?  A watch/ clock?  A radio?  A television?  A mobile telephone?  A refrigerator?  A table? A chair?  A bed with cotton/sponge/spring mattress?  An electric mitad?  Kerosene lamp/pressure lamp? | | | Yes No  Electricity?....................................1 2  A watch/ clock ?............................1 2  A radio?..........................................1 2  A television?...................................1 2  A mobile telephone?........................1 2  A refrigerator ?.................................1 2  A table ?...........................................1 2  A chair ?............................................1 2  Abed with cotton/sponge/spring mattress?............................................1 2  An electric mitad.................................1 2  A kerosene lamp/pressure lamp?...................................................1 2 | | | | | | | |  | |
| 220 | | Does your house have window? | | | 1. Yes  2. No | | | | | | | |  | |
| 221 | | Does any member of this household own any agricultural land? | | | 1.Yes  2.No | | | | | | | |  | |
| 222 | | How many (local units) of agricultural land do members of this household own? | | | ______________________local unit (Hectares, T imad) | | | | | | | |  | |
| 223 | | Which of the following your family own Milk cows or oxen?  Horses, donkeys or mules?  Goats or sheep?  Chickens?  Beehives? | | | Yes No  Milk cows or oxen………1 2  Horses or donkeys………1 2  Goats or sheep……………1 2  Chickens……………...… 1 2 Beehives…………………...1 2 | | | | | | | | 224 | |
| 224 | | If yes how many of them are your own? | | | Quantity  Milk cows_____ oxen _____  Horses _____donkeys_____ mules _____  Goats_____ sheep_____  Chickens_____  Beehives_____ | | | | | | | |  | |
| 225 | | Do you have access to media? | | | 1. Yes  2. No | | | | | | | | 226 | |
| 226 | | Which one of the following media does your family use? | | | 1. Radio  2. Television  3. Newspaper  4. internet  5. Others (specify__________________ | | | | | | | |  | |
| Part 3-Maternal growth Chart Knowledge | | | | | | | | | | | | | | |
| 301 | | | Do you heard about growth monitoring? | | | 1.Yes  2. N0 | | | | | | | | 302 |
| 302 | | | Do you know for what age groups growth monitoring service is given? | | | 1.less than two years  2. other  3.don,t know | | | | | | | |  |
| 303 | | | What is the Starting time for Growth monitoring? | | | 1.at birth  2. other  3.don,t know | | | | | | | |  |
| 304 | | | Interval Between GM visits? | | | 1.monthly  2. other  3.don,t know | | | | | | | |  |
| 305 | | | Do you know who to perform Growth monitoring? | | | 1.Health extension worker  2.other  3.don,t know | | | | | | | |  |
| 306 | | | Do you know where Growth monitoring service provision is performed? | | | 1.Health post  2.other  3.don,t know | | | | | | | |  |
| 307 | | | Do you know that taking your baby weigh regularly has benefit for your child? | | | 1.yes  2. no  3.don,t know | | | | | | | | 309 |
| 308 | | | What does it mean for a child when the curve on the growth chart is flattening? By using sample Growth chart | | | 1. Child is not growing well/has not gained enough weight  2. other  3. Don’t know | | | | | | | |  |
| 309 | | | What does it mean for a child when the curve on the growth chart is rising? By using sample Growth chart | | | 1.Child is growing well/has gained enough weight  2. other  3 .Don’t know | | | | | | | |  |
| 310 | | | What does it mean for a child when the curve on the growth chart is falling? By using sample Growth chart | | | 1.Child is not gaining weight from the previous growth monitoring and promotion session  2. other  3. Don’t know | | | | | | | |  |
| Part 4- Attitude Questions  Fill the boxes with “√“marks corresponding to your best choice based on the following Likert scale measures.  1= Strongly disagree (SD) 2= Disagree (D) 3= Neutral (N) 4= Agree (A) 5= Strongly agree (SA) | | | | | | | | | | | | | | |
| S.N | | | Questions | | | | | Strongly  Disagree | Disagree | Neutral | Agree | | Strongly  Agree | |
| 401 | | | When I go growth monitoring and promotion service I feel good about my child | | | | |  |  |  |  | |  | |
| 402 | | | When I go growth monitoring and promotion service I feel my child is healthy | | | | |  |  |  |  | |  | |
| 403 | | | When I go growth monitoring and promotion service I fell my child develop good academic performance | | | | |  |  |  |  | |  | |
| 404 | | | Do you think measuring your child weight is important? | | | | |  |  |  |  | |  | |
| 405 | | | Is bring their child to the growth monitoring and promotion service visits make you happy | | | | |  |  |  |  | |  | |
| 406 | | | Do you believe growth monitoring and promotion service is important to prevent malnutrition | | | | |  |  |  |  | |  | |
| 407 | | | Taking my child to growth monitoring and promotion service will take too much time | | | | |  |  |  |  | |  | |
| 408 | | | Taking my child to growth monitoring and promotion service doesn’t add any value to my child | | | | |  |  |  |  | |  | |
| 409 | | | Do you think growth monitoring and promotion is only for wasted children | | | | |  |  |  |  | |  | |
| 410 | | | Do you think growth monitoring and promotion is only for sick children | | | | |  |  |  |  | |  | |
| 411 | | | Taking my child to growth monitoring and promotion service will expose to evil eye | | | | |  |  |  |  | |  | |
| 412 | | | Do you think Growth monitoring is for screening children for food aid | | | | |  |  |  |  | |  | |
| Part 5: health system service factors | | | | | | | | | | | | | | |
| 501 | | | Is there health facilities available around your home | | | | 1. Yes  2. No | | | | | | |  |
| 402 | | | How far is the nearby health institution from your house? (In terms of hours it takes to reach on foot) | | | | ………………………………………hour …………………………………minutes | | | | | | |  |
| 503 | | | For what services do you usually take your children to the health facility?  (more than one response is possible) | | | | 1. weighting  2. nutrition advice  3. Immunization  4. Treatment of diseases  5. Vitamin A supplementation  6. Others(specify___________________ | | | | | | |  |
| 504 | | | Where do you get health service for your child usually? | | | | 1.health center  2.health post  3.private clinic  4.other (specify______________________ | | | | | | |  |
| 505 | | | Is growth monitoring and promotion service available for your child? | | | | 1.yes  2. no | | | | | | | 506 |
| 506 | | | What type of service offered in health facility? | | | | Yes No  Weighing…………………….1 2  Nutrition advice……………..1 2  Immunization ……………….1 2  Treatment of diseases………..1 2  vitamin A supplementation…..1 2 | | | | | | |  |
| 507 | | | utilization of ANC services | | | | 1.yes  2.No | | | | | | | 508 |
| 508 | | | If yes how often do you get the service? | | | | 1. >=4  2.<4 | | | | | | |  |
| 509 | | | utilization of PNC services | | | | 1.yes  2.No | | | | | | |  |
| 510 | | | counseling by health professionals about Growth monitoring | | | | 1.yes  2.No | | | | | | | 511 |
| 511 | | | If yes by what providers did you get counseling | | | | 1.Health Extension workers  2.Nurses  3.Midwives  4.Doctors  5.Teachers | | | | | | |  |
| 512 | | | How do you rate the services offered in the facility? | | | | 1. Good  2. Poor | | | | | | | 513  514 |
| 513 | | | If the answer is good, what is the reason? | | | | 1. Serving time less than 30 minutes,)  2. Services are regular  3. Services are always available  4. Others (specify____________________) | | | | | | |  |
| 514 | | | If the answer is poor, why? | | | | 1. Time taken is more than 1 hour,  2. Services are irregular,  3. Some services are not available  4. Others (specify______________________) | | | | | | |  |
| 515 | | | How do you rate the staffs’ performance? | | | | 1. Committed  2. Not committed | | | | | | | 516  517 |
| 516 | | | If the answer is committed, why? | | | | 1. Staffs always available to offer services  2. Staffs are very friendly  3.Others(specify ______________________) | | | | | | |  |
| 517 | | | If the answer is not committed, why? | | | | 1. Sometimes staffs are not available  2. Staffs are not very friendly  3. Others (specify _____________________) | | | | | | |  |
| 518 | | | Do you face any challenges when you take your child for weighing? | | | | 1. Yes  2. No | | | | | | | 519 |
| 519 | | | If yes, What Challenges do you face in taking your child for weighing? (Probe for all the challenges)  The healthy facility is far  It takes long to be served  Healthy workers are not always available  Inadequate time to take the child for GMP | | | | Yes No  The healthy facility is far…….……1 2  It takes long to be served …………1 2  Healthy workers are not always  available …………………………..1 2  Inadequate time to take the child  For GMP…………………..…….....1 2 | | | | | | |  |
